# Association of whole blood n-6 fatty acids with stunting in 2-to-6-year-old Northern Ghanaian children: A cross-sectional study

**DOI:** 10.1371/journal.pone.0193301

**Published:** 2018-03-01

**Authors:** Mary Adjepong, C. Austin Pickens, Raghav Jain, William S. Harris, Reginald A. Annan, Jenifer I. Fenton

**Affiliations:** 1 Department of Food Science and Human Nutrition, Michigan State University, East Lansing, Michigan, United States of America; 2 Sanford School of Medicine, University of South Dakota and Omega Quant Analytics, LLC, Sioux Falls, South Dakota, United States of America; 3 Kwame Nkrumah University of Science and Technology, Kumasi, Ghana; NIFES (National Institute of Nutrition and Seafood Research), NORWAY

## Abstract

In Northern Ghana, 33% of children are stunted due to economic disparities. Dietary fatty acids (FA) are critical for growth, but whether blood FA levels are adequate in Ghanaian children is unknown. The objective of this study was to determine the association between whole blood FAs and growth parameters in Northern Ghanaian children 2–6 years of age. A drop of blood was collected on an antioxidant treated card and analyzed for FA composition. Weight and height were measured and z-scores were calculated. Relationships between FAs and growth parameters were analyzed by Spearman correlations, linear regressions, and factor analysis. Of the 307 children who participated, 29.7% were stunted and 8% were essential FA deficient (triene/tetraene ratio>0.02). Essential FA did not differ between stunted and non-stunted children and was not associated with height-for-age z-score (HAZ) or weight-for-age z-score (WAZ). In hemoglobin adjusted regression models, both HAZ and WAZ were positively associated with arachidonic acid (p≤0.01), dihomo-gamma-linolenic acid (DGLA, p≤0.05), docosatetraenoic acid (p≤0.01) and the ratio of DGLA/linoleic acid (p≤0.01). These data add to the growing body of evidence indicating n-6 FAs are critical in childhood linear growth. Our findings provide new insights into the health status of an understudied Northern Ghanaian population.

## Introduction

Childhood growth stunting, deemed stunting, is a major nutritional challenge that affects over 165 million children globally [1]. Continents with the highest prevalence of stunting include Africa, Asia, and South America, where stunted and underweight children have a threefold higher risk of mortality [2] compared to well-nourished children. On the African continent, the Sub-Saharan region is most affected by stunting [1]. Observational studies in African countries such as Ghana report roughly 20% of children are stunted, with regional differences in Northern Ghana experiencing stunting rates over 30% [3]. Several fatty acid (FA) supplementation trials in Ghana reported increases in hemoglobin (Hb) levels of pregnant women and may support growth spurts in children [3, 4], however, there is a dearth of research investigating the relationship between circulating FA levels and growth in Ghana. FAs are important for childhood growth and development, and have roles in energy utilization, brain myelination [5], hormone synthesis [4], and FAs serve as substrates for signaling molecules.

FAs are synthesized *de novo*, except for the two essential FAs (EFAs): linoleic acid (LA, an omega-6 [n-6]) and alpha-linolenic acid (ALA, an omega-3 [n-3]) [5, 6]. Humans lack the delta-12 and delta-15 desaturase enzymes required to synthesize LA and ALA, thus, dietary intake is the primary source of EFAs. LA and ALA are elongated and desaturated to produce long chain polyunsaturated fatty acids (LC-PUFAs), and LC-PUFAs serve as substrates for eicosanoids such as prostaglandins, which are involved in cell differentiation and growth [7], and signal transduction [8]. The desaturation of LA and ALA is mediated by the delta-5 and delta-6 desaturase enzymes [9], while several elongases are involved in converting LA and ALA to LC-PUFAs.

The n-3 FAs and n-6 FAs have high affinity as substrates for elongase and desaturase enzymes but in their absence, omega-9 (n-9) FAs can serve as substrates for conversion to certain LC-PUFA species, notably the n-9 Mead acid [10, 11]. When an individual's diet is deficient of EFAs, oleic acid (C18:1n9), a non-essential n-9 FA, is converted to Mead acid (C20:3n9) [12]. Mead acid is then incorporated into phospholipids, cholesterol esters, triglycerides, and non-esterified free FAs [11, 13]. It is well accepted that those with EFA deficiency (EFAD), have higher levels of Mead acid and an elevated triene to tetraene ratio (T/T ratio), and the T/T ratio is defined as the ratio of Mead acid (3 double bonds, triene) to arachidonic acid (AA; 4 double bonds, tetraene) [14–17]. EFAD is defined by a T/T ratio > 0.02 in plasma samples [18, 19], and also established when Mead acid [19] levels are above 0.4% in red blood cells (RBCs) [17] and 0.21% in plasma [18].

Northern Ghanaian diets consist mainly of cereals and fruits, with intake of fats and proteins below adequate levels [11]. Additionally, only 36% of Ghanaian children between 6–36 month of age have fats added to their complementary food [20]. These low intakes of dietary fat may increase EFAD in infants and young children. In the Ghanaian diet, sources of LA include peanut and soybean oil, which are also high in ALA [21]. Fish, eggs, poultry, and whole grains are also good sources of EFAs [18, 22, 23]. In Ghana, malnutrition in infants and children below 5 years is mostly caused by inadequate complementary feeding practices [24] which can lead to insufficient EFA consumption.

Lipid-based supplementation has shown to increase linear growth in Ghanaian children [24, 25]. However, blood assessments of FA levels are underreported in Ghanaian children and, consequently, EFAD prevalence is poorly characterized. Given the importance of FAs in growth and development, and the high prevalence of stunting in Northern Ghana, the objectives of this study were to assess blood FA levels in 2-to-6-year-old Northern Ghanaian children and their associations with growth, and to characterize blood FA profiles for this population to aid future research and intervention studies. It was hypothesized that whole blood EFA levels in Ghanaian children would positively correlate with the growth measures weight-for-age (WAZ), weight-for-height (WHZ), height-for-age (HAZ) and BMI-for-age z-scores (BAZ).

## Subjects and methods

### Study setting

The study was conducted in the northern region of Ghana in the Savelugu-Nanton district [26]. The district covers 2022.6 sq. km with a population density of 68.9 persons per sq. km. The population of Savelugu-Nanton is 139,283 persons with 14,669 households. The average rainfall in the Savelugu-Nanton district is 600 mm. The district is also characterized by high temperatures with an average temperature of 34°C. The district is situated in the Savanna woodland that is capable of sustaining livestock, farming and the cultivation of crops such as rice, groundnuts, yams, cassava, maize, cowpea and sorghum. Over 80% of inhabitants are farmers. The main sources of water in the district are boreholes, rivers and streams, public taps, and pipe borne water. Though the primary source of water for taps and pipe borne water is the same, access to either categorizes households under different income levels, perhaps reflecting differences in hygiene and even nutritional status. Thatch is the main roofing material for housing (50.9%). Illiteracy level is high with 69% of all inhabitants 11 years and above having no education. Some common diseases in the district include malaria, gastroenteritis, upper respiratory tract infection, diarrhea, anemia, and pneumonia. There are three operational community health post zones that deliver health services to the people. The Savelugu-Nanton district was chosen for study as stunting levels are above the national average in the rural communities in this district [27] with overall district stunting level of 38.8% [28]. Additionally, it is one of the few areas in Northern Ghana with road access to rural communities.

### Ethical approval

This study was conducted according to the guidelines laid down in the Declaration of Helsinki and all procedures involving human subjects/patients were approved by the Institutional Review Board at Michigan State University (IRB # 16–557) and the Committee on Human Research Publication and Ethics, School of Medical Sciences, Kwame Nkrumah University of Science and Technology, Kumasi, Ghana (CHRPE/AP/236/16). The parent or caregiver of the participating child gave consent prior to the child’s participation. A script of the written consent was read and translated in Dagbani to the parents or caregivers of the children. The parents or caregivers thumb printed the consent document to give consent. They were assured that participation was voluntary and confidential, and that their information would remain anonymous.

### Sample size and subjects

Children (n = 307) between 2 to 6 years of age residing in 5 communities in the Savelugu-Nanton district were recruited for the study. The communities were Janjorikukuo, Pong Tamale, Kparigilanyi, Morglaa and Fazhini. A power analysis was conducted from the results of an earlier study that measured maternal and infant erythrocyte fatty acid intake [29], and the fatty acid variation reported was utilized to run an a-priori sample size calculation for multiple regression based on an estimated medium effect size of 0.5 and significance level p = 0.05. This indicated that 242 participants would yield statistical power of 80% [30]. 307 children were enrolled, raising the power to 90%. The exclusion criteria included sick and hospitalized children as well as children who were legally declared intellectually disabled. Data was collected in July 2016.

### Anthropometric measurements

Height of all participants was measured to the nearest 0.1cm with a stadiometer (Seca, USA). Weight was measured using a digital bathroom scale to the nearest 0.1kg (Camry, model number: EB9003, China). All measurements were repeated and averages were reported. The date of birth was recorded from the child’s health card or birth certificate. The sex of the child was also recorded.

### Blood fatty acid assessment

Blood spots (40ul) were collected on a dried blood spot card (DBS) as previously described by Jumbe et al., 2016 [4, 31]. A sterile single-use lancet was used in puncturing the tip of the middle finger to obtain drops of blood. The first drop of blood was wiped with a sterilized dry pad. The drops of blood were then collected onto the DBS cards. The cards were stored in a dry, cool environment and shipped to the USA for FA analysis at OmegaQuant Analytics, LLC (Sioux Falls, SD). The average time between sample collection and arrival in the US lab was 8 days. Upon arrival in the US lab, the samples were stored at –80°C for 5 days and then analyzed as previously described [29, 32, 33]. Briefly, a punch from the DBS card was combined with the derivatizing reagent [boron trifluoride in methanol (14%), toluene, and methanol (35:30:35 parts)], shaken and heated at 100°C for 45 minutes. Forty parts of both hexane and distilled water were added after the mixture had cooled. After vortexing briefly, the samples were spun to separate layers and an aliquot of the hexane layer that contained the FA methyl esters was extracted. FA analysis was performed as previously described [34–36]. Unless otherwise stated, whole blood FA proportions are expressed as a percent of total identified FAs.

### Hemoglobin and malaria status

Additional drops of blood from the same puncture site were used to assess hemoglobin concentration using a HemoCue photometer (HemoCue 301, Angelholm, Sweden), and malaria status using an antigen-based malaria rapid diagnostic test kit (Standard diagnostic Inc., Korea).

### Data reduction and statistical analyses

Z-scores were calculated for the growth parameters HAZ, WAZ, and WHZ using WHO Anthro v3.2.2 igrowup package for R [37], to calculate z-scores for children < 5 years of age, and WHO Anthro Plus [38] for children ≥ 5. Means and standard deviations were calculated for descriptive analysis. Stunting percentages were calculated based on the WHO standard population and definitions of moderate and severe stunting, wasting, and underweight [39]. FAs were expressed as percent composition of total blood FAs. Mean and standard deviations were calculated for blood FA composition. Total n-3 FA proportions were calculated as ∑ [ALA+ eicosapentaenoic acid (EPA) + docosapentaenoic n-3 (DPA n-3) + docosahexaenoic acid (DHA)]; total n-6 FA proportions were calculated as ∑ [LA + linoelaidic + eicosadienoic (EDA) + dihomo-gamma-linolenic (DGLA) + AA + docosatetraenoic (DTA) + docosapentaenoic n-6 (DPA n-6)]; total n-9 FA proportions were calculated as ∑ [oleic + elaidic + eicosenoic + Mead + nervonic]; total saturated FA proportions were calculated as ∑ [myristic + palmitic + stearic + arachidic + behenic + lignoceric]; total monounsaturated FA (MUFA) proportions were calculated as ∑ [palmitoleic + oleic + palmitelaidic + nervonic + elaidic + eicosenoic]; total polyunsaturated FA (PUFA) proportions were calculated as ∑ [total n-3 + total n-6]. T/T ratio was calculated from the ratio of Mead acid and AA [37]. Product-to-precursor ratios were calculated to estimate PUFA metabolism [40] as follows: EDA/LA to estimate elongase activity, GLA/LA and AA/DGLA to estimate desaturase activity, and DGLA/LA to estimate combined elongase and desaturase activity.

All statistical analyses were conducted using software R (R version 3.4.0). Correlations between participant characteristics, anthropometric measurements, and blood FAs were assessed using spearman correlations and graphically displayed using the R package *corrplot* [41]. Normal probability plots were assessed to verify the validity of regressions. Regression formulas consisted of either the dependent variable HAZ or WAZ, and models were adjusted for each FA and Hb levels (i.e., HAZ = FA + Hb or WAZ = FA + Hb). Hemoglobin was selected as a covariate since it was significantly associated with HAZ and WAZ (p ≤0.01). Regression models were adjusted for Hb and not adjusted for sex as there were few significantly different fatty acids (FAs) between sexes and regression values were unaffected when evaluated with sex adjustment. P-values were considered significant if p≤0.05. Exploratory factor analysis was carried out using the *psych* package [42]. Briefly, scree plot was used to determine four factors [43]. Palmitelaidic, linoelaidic, and elaidic acids were omitted from the analysis as they were not highly correlated with any other FAs (r<0.3). Varimax rotation was used for orthogonal transformation of the factor loading matrix. FAs correlated with factors r≥0.5 were considered strongly correlated with the factor, regardless of sign. Factor loading scores were generated for each child and used to calculate regressions for each factor. The regressions were HAZ or WAZ = Hb + Factor.

## Results

### Subject characteristics

Demographic information is presented in Table 1. In this study, the median age of all 307 children was 46.8 months, with the youngest and oldest being 24.0 and 70.8 months, respectively. There were more males (52.1%) than females (47.9%) in the study. The median height of participants was 96.1 cm, and the median weight of participants was 13.5 kg. Hb levels ranged from 8.4 g/dl to 13.6 g/dl, malaria positivity was detected in 2.9% of the children, and all children were breastfed as infants with 94% of them having been breastfed until 20 months of age (S1 Original Data). The median HAZ, WAZ, WHZ and BAZ were -1.31, –1.16, -0.45, and –0.33, respectively. None of the participant characteristics differed by sex (Table 1). The standard deviations of the HAZ, WAZ, and WHZ distributions were relatively constant and close to the expected value of 1.0 (range: 0.78–1.14, S1 Original Data). According to the WHO criteria [42], 29.3% of the children were stunted and 15% were underweight (Table 2). Approximately, 3.5% were categorized as wasting, or had a low BMI for their age.

**Table 1:** Sex differences are not associated with characteristics of participants^a^.

	Median (Q1, Q3)			
	Overall n = 307	Male n = 160	Female n = 147	p-value^b^
Age (mo)	46.8 (37.2, 57.0)	48.0 (37.2, 57.6)	45.6 (37.2, 56.4)	0.73
Height (cm)	96.1 (80.0, 102)	96.6 (90.7, 103)	95.6 (89.1, 101)	0.13
Weight (kg)	13.5 (12.2, 15.5)	13.9 (12.6, 15.6)	13.3 (11.9, 15.3)	0.07
HAZ	-1.30 (-2.10, -0.69)	-1.32 (-2.10, -0.52)	-1.30 (-2.10, -0.85)	0.50
BAZ	-0.33 (-0.91, 0.11)	-0.24 (-0.96, 0.11)	-0.37 (-0.80, 0.09)	0.72
WAZ	-1.20 (-1.70, -0.52)	-1.20 (-1.70, -0.44)	-1.10 (-1.70, -0.58)	0.83
WHZ	-0.45 (-1.00, 0.02)	-0.39 (-1.10, 0.02)	-0.52 (-0.91, -0.05)	0.40
Hb (g/dL)	11.0 (10.3, 11.7)	11.0 (10.3, 11.7)	10.9 (10.4, 11.7)	0.60

^a^The WHO definitions of moderate and severe stunting, wasting, underweight and malnutrition were applied to the data [42].

HAZ, height-for-age z-score; BAZ, BMI-for-age z-score; WAZ, weight-for-age z-score; WHZ, weight-for-height z-score; Hb, hemoglobin.

^b^Wilcoxon-Mann-Whitney test was conducted to assess sex differences, p-values presented.

**Table 2:** Nutrition and growth status of the children^a^.

	Based on	Severe	Moderate	Unaffected
		(<-3SD)	(≤-2SD)	
Stunting	HAZ	6.07%	23.6%	70.3%
Malnutrition	BAZ	0.00%	3.19%	96.8%
Underweight	WAZ	1.60%	13.4%	85.0%
Wasting	WHZ	0.00%	3.50%	96.5%

^a^The WHO definitions of moderate and severe stunting, wasting, underweight and malnutrition were applied to the data [42].

SD, standard deviation; HAZ, height-for-age z-score; BAZ, BMI-for-age z-score; WAZ, weight-for-age z-score; WHZ, weight-for-height z-score.

### Fatty acid levels in whole blood

The median, first, and third quartiles comparing selected FAs of stunted (HAZ≤-2) and non-stunted children are shown in Table 3, and values for all FAs analyzed in our study are presented in S1 Table. Approximately 8% of all children in the study had a whole blood T/T ratio greater than 0.02 and 6.8% of the study population had whole blood Mead acid levels above 0.21%. Oleic acid and total n-9 were significantly higher in stunted children, while DHA, the omega-3 index, and total n-3, and AA and DTA were all higher in non-stunted children. There was no significant difference for the T/T ratio or Mead acid between boys and girls (S1 Original Data).

**Table 3:** Median (Q1, Q3) of select fatty acid proportions in whole blood^a^.

Class	Fatty acid	Overall	Stunted	Non-stunted	p-value^b^
n-9	Oleic	20.8 (19.5, 22.6)	21.1 (20.1, 23.7)	20.6 (19.5, 22.2)	**≤0.05**
	Elaidic	0.17 (0.14, 0.23)	0.17 (0.14, 0.25)	0.17 (0.14, 0.23)	0.64
	Eicosenoic	0.31 (0.27, 0.39)	0.31 (0.27, 0.36)	0.31 (0.27, 0.40)	0.61
	Mead	0.13 (0.10, 0.16)	0.12 (0.10, 0.15)	0.13 (0.11, 0.17)	0.12
	Nervonic	0.72 (0.59, 0.91)	0.71 (0.57, 0.92)	0.73 (0.60, 0.90)	0.32
	Total n-9^c^	21.9 (20.7, 23.6)	22.2 (21.3, 24.5)	21.7 (20.5, 23.4)	**≤0.05**
n-3	ALA	0.16 (0.11, 0.21)	0.16 (0.11, 0.24)	0.15 (0.11, 0.21)	0.75
	EPA	0.18 (0.13, 0.24)	0.18 (0.13, 0.24)	0.18 (0.13, 0.24)	0.89
	DPA n-3	0.55 (0.47, 0.67)	0.54 (0.46, 0.67)	0.56 (0.47, 0.67)	0.72
	DHA	2.53 (2.18, 2.96)	2.42 (2.09, 2.76)	2.60 (2.24, 3.03)	**≤0.01**
	Total n-3^d^	3.47 (3.08, 3.95)	3.30 (2.98, 3.78)	3.51 (3.12, 4.00)	**≤0.05**
	O3I	2.70 (2.36, 3.17)	2.58 (2.29, 3.03)	2.74 (2.41, 3.20)	**≤0.01**
n-6	LA	20.7 (19.4, 21.7)	20.8 (19.7, 22.1)	20.6 (19.2, 21.5)	0.14
	GLA	0.15 (0.12, 0.20)	0.16 (0.12, 0.19)	0.15 (0.11, 0.20)	0.74
	EDA	0.29 (0.24, 0.33)	0.28 (0.24, 0.34)	0.29 (0.25, 0.33)	0.41
	DGLA	1.36 (1.18, 1.52)	1.35 (1.18, 1.49)	1.37 (1.19, 1.54)	0.32
	AA	11.0 (9.94, 11.9)	10.8 (9.60, 11.4)	11.2 (10.0, 11.9)	**≤0.01**
	DTA	1.68 (1.44, 1.92)	1.62 (1.36, 1.80)	1.72 (1.48, 1.95)	**≤0.01**
	DPA n-6	0.58 (0.47, 0.69)	0.54 (0.46, 0.68)	0.59 (0.48, 0.70)	0.06
	Total n-6^e^	36.0 (34.4, 37.4)	35.8 (34.5, 36.7)	36.1 (34.3, 37.6)	0.10
Ratios	GLA/LA	0.01 (0.01, 0.01)	0.01 (0.01, 0.01)	0.01 (0.01, 0.01)	0.93
	EDA/LA	0.01 (0.01, 0.02)	0.01 (0.01, 0.02)	0.01 (0.01, 0.02)	0.15
	DGLA/LA	0.07 (0.06, 0.08)	0.07 (0.06, 0.07)	0.07 (0.06, 0.08)	0.11
	AA/DGLA	8.05 (7.21, 8.99)	7.88 (7.04, 8.79)	8.11 (7.31, 9.08)	0.19

^a^Values represent blood fatty acid (FA) % composition.

Stunted defined by height-for-age z-score (HAZ)≤-2. n-9, omega-9

ALA, alpha-linolenic acid; EPA, eicosapentaenoic acid; DPA n-3, omega-3 docosapentaenoic acid; DHA, docosahexaenoic acid; n-3, omega-3; O3I, omega-3 index; LA, linoleic acid; GLA, gamma-linolenic acid; EDA, eicosadienoic acid; DGLA, dihomo-gamma-linolenic acid; AA, arachidonic acid; DTA, docosatetraenoic acid; DPA n-6, omega-6 docosapentaenoic acid; n-6, omega-6.

^b^P-value from Wilcoxon-Mann-Whitney test comparing stunted and non-stunted children.

^c^Total n-9 includes oleic, elaidic, eicosenoic, Mead, and Nervonic.

^d^Total n-3 includes ALA, EPA, DPA n-3, and DHA.

^e^Total n-6 includes LA, linoelaidic, GLA, EDA, DGLA, AA, DTA, and DPA n-6.

### Correlations between fatty acids and growth parameters

**Fig 1:**
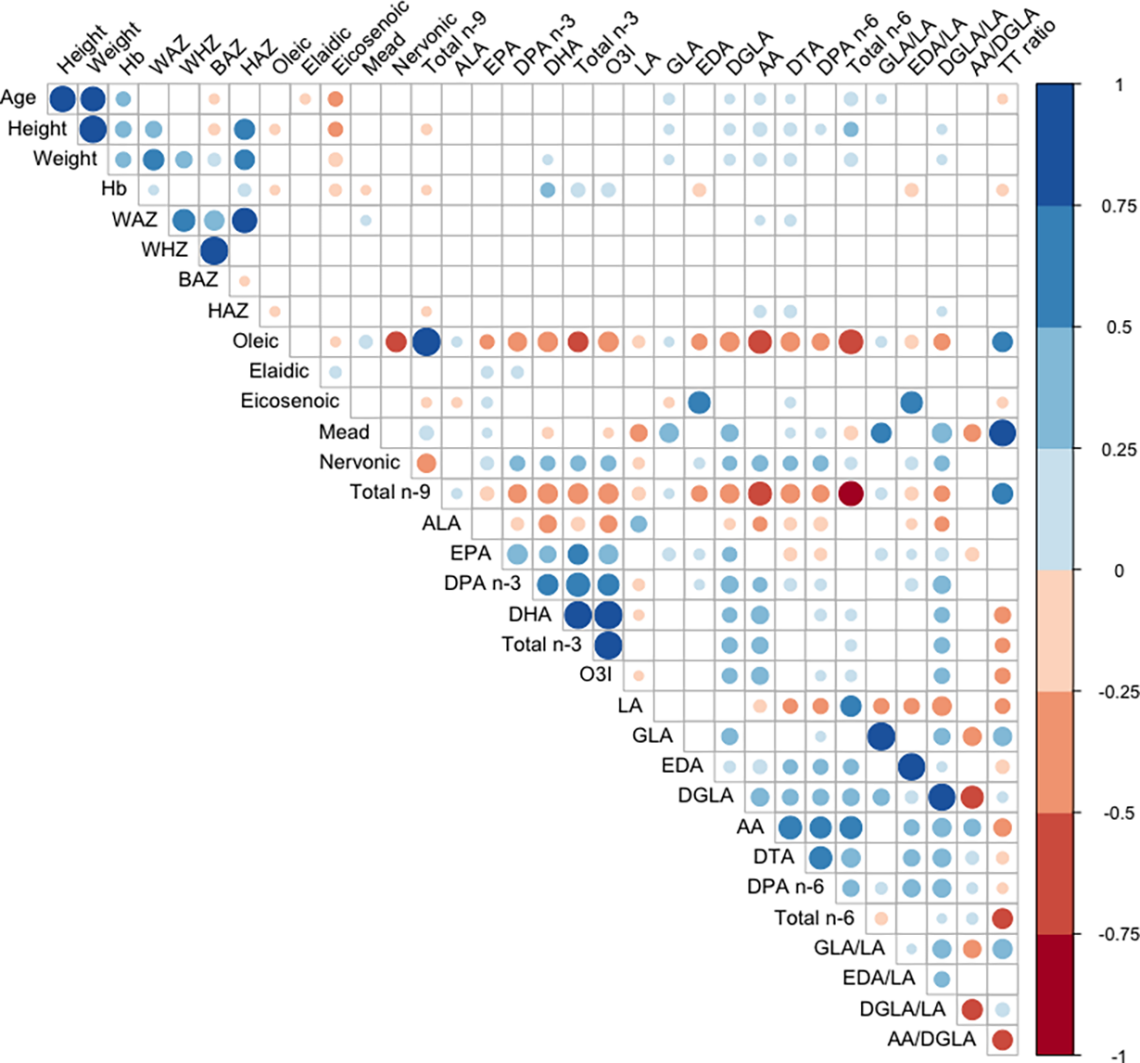
Spearman correlations between participant characteristics, anthropometric measurements, and selected blood FA levels.^a^ ^a^Spearman correlation matrix displays r correlation coefficients represented as circles, where large circles represent values closer toward 1 or -1 and smaller circles represent values closer toward 0.1 and -0.1. Blue shades denote positive r correlation coefficients, while red shades denote negative r correlation coefficients. Only results with p≤0.05 are displayed, thus, empty boxes were not significant. FA, fatty acid; Hb, hemoglobin; WAZ, weight-for-age z-score; WHZ, weight-for-height z-score; BAZ, BMI-for-age z-score; HAZ, height-for-age z-score; n-9, omega-9; ALA, alpha-linolenic acid; EPA, eicosapentaenoic acid; DPA n-3, omega-3 docosapentaenoic acid; DHA, docosahexaenoic acid; n-3, omega-3; O3I, omega-3 index; LA, linoleic acid; GLA, gamma-linolenic acid; EDA, eicosadienoic acid; DGLA, dihomo-gamma-linolenic acid; AA, arachidonic acid; DTA, docosatetraenoic acid; DPA n-6, omega-6 docosapentaenoic acid; n-6, omega-6. Total n-9 includes oleic, elaidic, eicosenoic, Mead, and Nervonic. Total n-3 includes ALA, EPA, DPA n-3, and DHA. Total n-6 includes LA, linoelaidic, GLA, EDA, DGLA, AA, DTA, and DPA n-6.

Spearman correlations were calculated for participant characteristics, anthropometric measurements, and selected FAs (Fig 1), and results for all FAs analyzed in this study are presented in S2 Table. HAZ was positively correlated with AA (p≤0.01) and DTA (p≤0.01). HAZ was negatively correlated with total n-9 FAs (p≤0.05). WAZ was positively correlated with AA (p≤0.05) and DTA (p≤0.05). Interestingly, height and weight were positively correlated with the ratio of DGLA/LA (pc0.05), but were not significantly associated with either GLA/LA or EDA/LA. No significant associations were observed between the blood FA levels and BAZ or WHZ.

### Relationships or associations between fatty acids and growth parameters

Table 4 shows the results of the regression analysis for HAZ and WAZ by selected FAs. Since the results of models adjusted for Hb and Hb + Sex were similar (S1 Original Data), only Hb adjusted models are presented. There was a significant, positive relationship between HAZ and AA (p≤0.01), DGLA (p≤0.05), DTA (p≤0.01), and DGLA/LA (p≤0.01). In addition, WAZ was positively associated with stearic acid (p≤0.05), AA (p≤0.01), DGLA (p≤0.05), DTA (p≤0.01), and DGLA/LA (p≤0.01). Mead acid was positively associated with both HAZ and WAZ. The n-3 PUFAs were not associated with any of the growth parameters. No significant associations were observed between any of the FAs and WHZ or BAZ. A table of all analyzed FA regressions can be found in Supplementary Materials (S3 Table).

**Table 4:** Regression results between HAZ, WAZ, and selected fatty acids (model: HAZ = fatty acid + hemoglobin; WAZ = fatty acid + hemoglobin; HAZ = ratio + hemoglobin; WAZ = ratio + hemoglobin)^a^.

		HAZ		WAZ	
Class	Fatty acid	Beta ± SE	p-value	Beta ± SE	p-value
n-9	Oleic	-0.04 ± 0.02	0.10	-0.03 ± 0.02	0.14
	Elaidic	-0.02 ± 0.33	0.94	-0.04 ± 0.25	0.87
	Eicosenoic	-0.35 ± 0.60	0.56	-0.01 ± 0.46	0.98
	Mead	2.48 ± 1.21	**≤0.05**	2.08 ± 0.93	**≤0.05**
	Nervonic	0.13 ± 0.29	0.65	0.14 ± 0.23	0.53
	Total n-9^b^	-0.04 ± 0.02	0.10	-0.03 ± 0.02	0.15
n-3	ALA	-0.67 ± 0.56	0.23	-0.32 ± 0.43	0.45
	EPA	0.07 ± 0.25	0.77	0.17 ± 0.19	0.36
	DPA n-3	0.03 ± 0.37	0.93	0.20 ± 0.28	0.48
	DHA	0.08 ± 0.10	0.43	0.09 ± 0.08	0.26
	Total n-3^c^	0.04 ± 0.07	0.62	0.06 ± 0.06	0.27
	O3I	0.06 ± 0.08	0.47	0.08 ± 0.06	0.23
n-6	LA	-0.06 ± 0.03	0.07	-0.04 ± 0.03	0.11
	GLA	-0.19 ± 0.95	0.84	-0.21 ± 0.73	0.78
	EDA	-1.48 ± 0.94	0.12	-0.80 ± 0.72	0.27
	DGLA	0.51 ± 0.25	**≤0.05**	0.39 ± 0.19	**≤0.05**
	AA	0.12 ± 0.04	**≤0.01**	0.08 ± 0.03	**≤0.01**
	DTA	0.50 ± 0.17	**≤0.01**	0.39 ± 0.13	**≤0.01**
	DPA n-6	0.56 ± 0.38	0.14	0.18 ± 0.29	0.54
	Total n-6^d^	0.03 ± 0.03	0.18	0.02 ± 0.02	0.26
Ratios	GLA/LA	4.19 ± 17.7	0.81	0.54 ± 13.6	0.97
	EDA/LA	-13.8 ± 18.3	0.45	-6.52 ± 14.0	0.64
	DGLA/LA	11.7 ± 4.39	**≤0.01**	8.34 ± 3.38	**≤0.01**
	AA/DGLA	0.02 ± 0.04	0.68	0.00 ± 0.03	0.92

^a^Models were adjusted for Hb.

Beta ± standard error (SE) presented.

HAZ, height-for-age z-score; WAZ, weight-for-age z-score; n-9, omega-9; n-3, omega-3; ALA, alpha-linolenic acid; EPA, eicosapentaenoic acid; DPA n-3, omega-3 docosapentaenoic acid; DHA, docosahexaenoic acid; O3I, omega-3 index; n-6, omega-6; LA, linoleic acid; GLA, gamma-linolenic acid; EDA, eicosadienoic acid; DGLA, dihomo-gamma-linolenic acid; AA, arachidonic acid; DTA, docosatetraenoic acid; DPA n-6, omega-6 docosapentaenoic acid.

^b^Total n-9 includes oleic, elaidic, eicosenoic, Mead, and Nervonic acid.

^c^Total n-3 includes ALA, EPA, DPA n-3, and DHA.

^d^Total n-6 includes LA, linoelaidic, GLA, EDA, DGLA, AA, DTA, and DPA n-6.

### Factor analysis

Exploratory factor analysis was conducted to identify FA patterns that may be related to stunting. Scree Plot analysis indicated that four factors should be extracted. Factor loadings (Table 5) show the correlation between individual FAs and the respective factor. Individual factor loadings for each factor were regressed against HAZ and WAZ (Table 6). Factor 1 was significantly associated with HAZ and WAZ (p≤0.01). Several n-6 fatty acids, including AA, DGLA, and DTA were highly correlated with this factor. Factors 2–4 were not significantly associated with either HAZ or WAZ.

**Table 5:** Factor analysis of fatty acids^a^.

Fatty acid	Factor 1	Factor 2	Factor 3	Factor 4
AA	**0.89**	-0.09	0.08	0.14
DTA	**0.81**	0.07	0.13	-0.15
DPA n-6	**0.72**	0.27	0.09	-0.11
Stearic	**0.64**	-0.06	0.24	0.07
DGLA	**0.59**	0.31	-0.15	0.20
Palmitic	**-0.55**	0.27	-0.25	0.04
Oleic	**-0.69**	0.22	-0.08	-0.49
Palmitoleic	-0.23	**0.80**	0.10	0.12
Myristic	-0.38	**0.66**	0.04	0.09
GLA	0.05	**0.65**	-0.19	-0.06
Mead	0.18	**0.62**	-0.04	-0.16
LA	-0.19	**0.62**	-0.20	-0.02
Arachidic	-0.04	-0.17	**0.78**	-0.26
Eicosenoic	-0.05	0.13	**0.74**	0.18
Behenic	0.35	-0.29	**0.73**	-0.01
Lignoceric	0.49	-0.25	**0.54**	0.08
Nervonic	0.44	-0.05	**0.50**	0.21
DPA n-3	0.12	0.12	0.06	**0.87**
DHA	0.23	-0.07	-0.01	**0.82**
EPA	-0.22	-0.06	0.08	**0.81**
ALA	-0.35	-0.25	-0.23	-0.25
EDA	0.17	0.15	0.47	0.08

^a^Varimax rotated factor-loading matrix generated using the R-package psych.

Factors named based on majority of highly correlated FAs. Numbers displayed represent each FA correlation with its respective factor. Correlations ≥0.50 are bolded.

AA, arachidonic acid; DTA, docosatetraenoic acid; DPA n-6, omega-6 docosapentaenoic acid; DGLA, dihomo-gamma-linolenic acid; GLA, gamma-linolenic acid; LA, linoleic acid; DPA n-3, omega-3 docosapentaenoic acid; DHA, docosahexaenoic acid; EPA, eicosapentaenoic acid; ALA, alpha-linolenic acid; EDA, eicosadienoic acid.

**Table 6:** HAZ and WAZ regressed on calculated factors (HAZ or WAZ = Factor + Hb)^a^.

	HAZ		WAZ	
	Beta	p-value	Beta	p-value
**Factor 1**	0.18	**≤0.01**	0.13	**≤0.01**
**Factor 2**	0.03	0.60	0.01	0.82
**Factor 3**	-0.09	0.18	-0.01	0.91
**Factor 4**	0.01	0.86	0.02	0.65

^a^HAZ, height for age z-score; WAZ, weight for age z-score; Hb, hemoglobin.

## Discussion

The purpose of this study was to characterize the whole blood FA levels of Northern Ghanaian children and determine FA associations with growth parameters. Based on Mead acid levels and the T/T ratio, we found that EFAD was low in this population, however, the prevalence of stunting was high (29%). Furthermore, 6.7% of all participants had Mead acid levels above 0.21%, and 8.0% had a T/T ratio greater than 0.02, both lower than previously reported in Tanzania [42]. Interestingly, Mead acid was positively associated with both HAZ and WAZ despite low EFAD. In regression analyses, n-6 LC-PUFAs were inversely associated with stunting, and T/T ratio and total n-3 FAs were not significantly associated with any of the growth parameters. Taken together, our results indicate that whole blood n-6 LC-PUFAs levels are inversely associated with growth stunting, and even though EFAD was low, Mead acid levels were positively associated with growth parameters in this population of Northern Ghanaian children.

The levels of EFAs (i.e., ALA and LA) did not differ between stunted and non-stunted children and were not associated with measures of linear growth. The n-3 LC-PUFAs were not associated with growth in regression analyses, although DHA, total n-3, and the omega-3 index were significantly lower in stunted children compared to non-stunted children. Although whole blood LA levels did not differ, n-6 LC-PUFAs such as AA, DTA, and DGLA were low in stunted children. As DGLA, AA, DTA, and total n-6 FA levels increased in the blood, there was an increase in HAZ, thus, a decrease in stunting. Overall these findings are consistent with previous reports in Tanzanian children [37] and support evidence that n-6 FAs are important in linear growth [44].

Dietary intake of PUFAs is reflective of whole blood PUFA levels [45], therefore, increasing dietary intake of PUFAs in these children may increase whole blood PUFA levels, improve growth parameters, and reduce stunting. AA status is positively correlated with first year growth in preterm infants [46], and AA can regulate gene expression related to cytokine production and osteoclast differentiation [45]. Dietary AA also positively correlates with plasma insulin-like growth factor I concentrations [45] which increases hypertrophic cell size and linear growth [47], and is a main predictor of height velocity in children. The mechanisms by which n-6 FAs affect growth and development include serving as substrates for the synthesis of ligands in signal transduction pathways [37]. For instance, AA-derived eicosanoids have key roles in normal growth and development, with prostaglandin E2 functioning in hormone regulation of bone development [44, 45, 48, 49].

Since the n-6 PUFAs DGLA, AA, and DTA were significantly associated with growth parameters, product-to-precursor ratios were assessed to investigate PUFA metabolism. As mentioned LA levels did not differ in regression or dichotomous analyses. The conversion of LA (C18:2 n-6) to DGLA (C20:3 n-6) occurs by: 1) LA elongation to form EDA, then EDA desaturation to form DGLA; or 2) LA desaturation to form GLA, then GLA is elongation to form DGLA [50]. The ratios of GLA/LA or EDA/LA were not significantly associated with growth parameters, however, the ratio of DGLA/LA was significantly associated with growth parameters. The *de novo* conversion of LA to DGLA utilizes the elongation of very long chain fatty acid protein 5 (ELOVL-5) and delta-6-desaturase enzyme, while the conversion of DGLA (C20:3) to AA (C20:4) utilizes the delta-5-desaturase enzyme. The ratio of AA/DGLA was not significantly associated with growth parameters. It is possible in this population of Northern Ghanaian children that PUFA metabolism is altered through several enzymes involved in FA metabolism. What remains unclear is whether these observations are due to altered PUFA metabolism, lower dietary intakes of PUFA, or both.

Gene-diet interactions are known to potentially modulate enzyme activities of some desaturases that are involved in FA metabolism [51]. Fatty acid desaturase-1 (FADS1) and FADS2 genes that encode delta-5-desaturase and delta-6-desaturase, respectively [52], have been hypothesized to be under selective pressure by extreme PUFA diets. The hallmark paper by Fumagalli et al. (2015) reported that isolated populations with extreme diets in varying PUFA composition coped by physiologically adapting gene variants in FADS enzymes [53]. More interestingly, is these FADS gene variants were found to also significantly influence height and weight. We report Mead acid, a PUFA well recognized to accumulate under conditions of EFAD, was positively associated with HAZ and WAZ. This result was unexpected. Mead acid did not differ between stunted and non-stunted children, nor did LA or T/T ratio. One possible explanation is this rural population in Northern Ghana, over the millennia, may have physiologically adapted to EFAD and diets low in PUFAs, similar to the findings of Fumagalli et al. As previously mentioned we report that EFAD was low in this population, despite a prevalence of stunting at 29%. When individuals are deficient in dietary LA, oleic acid is desaturated by delta-6-desaturase, elongated by ELOVL5, and finally desaturated by delta-5-desaturase to form Mead acid [32]. We report the ratio of DGLA/LA was significantly associated with both HAZ and WAZ, and the *de novo* conversion of DGLA/LA is also dependent on delta-6-desaturase and ELOVL-5. Furthermore, in these children, Mead acid and the ratio of DGLA/LA were highly correlated (Fig 1 and S2 Table). Future research should investigate FADS gene variants in these children to determine if our findings are due to altered metabolism at the biochemical level or due to altered dietary intake.

The purpose of this study was to assess blood FA levels in 2-to-6-year-old Northern Ghanaian children and associations with growth parameters. This study cannot be generalized to the entire Ghanaian population since the study was performed in one village and dietary intake of foods can differ across Ghana. The authors acknowledge the children were not required to fast prior to whole blood collection, blood was collected throughout the day, and these factors may have added variability to our results. However, this variability is expected to be minimal since children in this village consume similar, low-fat meals compared to children in other populations. We did not measure indices of body fat such as mid-upper arm circumference or body composition. In addition, we do not have data on nutrient intake in this population. Nutritional deficiencies aside from EFAD can lead to poor growth in the children. For example, zinc can also affect FA metabolism, and we did not measure zinc. We acknowledge that product-to-precursor ratios provide an indirect estimation of enzyme activity and may not fully reflect biochemical activity. The authors speculate our Mead acid finding may be related to altered PUFA metabolism at the enzymatic level, and conducting this analysis was outside the scope of our current study. Therefore, future researchers should investigate FADS enzyme gene variants in this region of Northern Ghana.

To our knowledge this is the first study to assess whole blood FAs in Ghanaian children 2–6 year olds. This study utilized biomarkers of FA status rather than food intake questionnaires to study the associations between FA status and growth. The study was large enough to detect an association between growth metrics and FA levels. Additionally, the use of a validated dried blood spot collection and blood transport system made the study logistically easier to conduct, and the method was also successfully used in a similar study in Tanzania [54]. Our findings add to the growing body of evidence indicating n-6 FAs play a crucial role in linear growth. These data provide new insights into the health of rural Northern Ghanaian children and valuable information for potential intervention studies attempting to combat stunting via nutrient supplementation.

## Supporting information

S1 Table Median (Q1, Q3) of fatty acid proportions in whole blood. Values represent blood fatty acid (FA) % composition.
(DOCX)

S2 Table Significant (p≤.05) correlations between all FAs and growth parameters. Correlations for all FA and growth parameters shown that are significant.
(DOCX)

S3 Table Regression results between HAZ, WAZ, and fatty acids (model: HAZ = fatty acid + hemoglobin; WAZ = fatty acid + hemoglobin; HAZ = ratio + hemoglobin; WAZ = ratio + hemoglobin). Model is not adjusted for sex as there were few significantly different FAs between sexes and regression values were essentially unaffected when evaluated with sex adjustment.
(DOCX)

S1 Original Data FA and growth data of Northern Ghana children. Raw data used for all analyses.
(CSV)

## Abbreviations

AA: arachidonic acid
ALA: alpha-linolenic acid
BAZ: BMI-for-age z-score
BMI: body mass index
DBS: dried blood spot
DGLA: dihomo-gamma-linolenic acid
DHA: docosahexaenoic acid
DPA: docosapentaenoic acid
DTA: docosatetraenoic acid
EDA: eicosadienoic acid
EFA: essential fatty acid
EFAD: essential fatty acid deficiency/deficient
ELOVL-5: elongation of very long chain fatty acid protein 5
EPA: eicosapentaenoic acid
FA: fatty acid
FADS: fatty acid desaturase
GLA: gamma-linolenic acid
HAZ: height-for-age z-score
Hb: hemoglobin
LA: linoleic acid
LC-PUFA: long chain poly-unsaturated fatty acids
n-3: omega-3
n-6: omega-6
O3I: omega-3 index
RBC: red blood cell
SD: standard deviation
SE: standard error
T/T: triene to tetraene ratio
WAZ: weight-for-age z-score
WHZ: weight-for-height z-score
