# Supplementary material for: Association of whole blood n-6 fatty acids with stunting in 2-to-6-year-old Northern Ghanaian children: A cross-sectional study
Source: PLoS One. 2018 Mar 1;13(3):e0193301. doi: 10.1371/journal.pone.0193301 (PMC5832227; doi:10.1371/journal.pone.0193301)
Supplement: S1 Table — Values represent blood fatty acid (FA) % composition. (DOCX) [file pone.0193301.s001.docx]

S1 Table: Median (Q1, Q3) of fatty acid proportions in whole blood.^a^

| **Class** | **Fatty acid** | **Overall** | **Stunted** | **Non-stunted** | **p-value^b^** |
| --- | --- | --- | --- | --- | --- |
| SFA | Myristic | 0.17 (0.12, 0.27) | 0.19 (0.13, 0.29) | 0.16 (0.11, 0.26) | 0.25 |
|  | Palmitic | 21.3 (20.3, 22.3) | 21.4 (20.8, 22.4) | 21.2 (20.2, 22.2) | 0.21 |
|  | Stearic | 13.5 (12.6, 14.3) | 13.4 (12.4, 14.2) | 13.6 (12.8, 14.3) | 0.08 |
|  | Arachidic | 0.35 (0.31, 0.39) | 0.35 (0.31, 0.40) | 0.35 (0.31, 0.39) | 0.35 |
|  | Behenic | 0.86 (0.72, 0.98) | 0.84 (0.69, 0.99) | 0.87 (0.73, 0.98) | 0.33 |
|  | Lignoceric | 1.24 (1.04, 1.43) | 1.21 (0.98, 1.42) | 1.25 (1.06, 1.45) | 0.40 |
|  | Total SFA^c^ | 37.5 (36.8, 38.5) | 37.4 (36.8, 38.4) | 37.5 (36.8, 38.5) | 0.45 |
|  |  |  |  |  |  |
| n-9 | Oleic | 20.8 (19.5, 22.6) | 21.1 (20.1, 23.7) | 20.6 (19.5, 22.2) | **≤0.05** |
|  | Elaidic | 0.17 (0.14, 0.23) | 0.17 (0.14, 0.25) | 0.17 (0.14, 0.23) | 0.64 |
|  | Eicosenoic | 0.31 (0.27, 0.39) | 0.31 (0.27, 0.36) | 0.31 (0.27, 0.40) | 0.61 |
|  | Mead | 0.13 (0.10, 0.16) | 0.12 (0.10, 0.15) | 0.13 (0.11, 0.17) | 0.12 |
|  | Nervonic | 0.72 (0.59, 0.91) | 0.71 (0.57, 0.92) | 0.73 (0.60, 0.90) | 0.32 |
|  | Palmitoleic | 0.32 (0.22, 0.45) | 0.35 (0.25, 0.47) | 0.31 (0.21, 0.42) | 0.06 |
|  | Total n-9^d^ | 21.9 (20.7, 23.6) | 22.2 (21.3, 24.5) | 21.7 (20.5, 23.4) | **≤0.05** |
|  |  |  |  |  |  |
| n-7 | Palmitelaidic | 0.02 (0.01, 0.04) | 0.03 (0.02, 0.04) | 0.02 (0.01, 0.04) | 0.14 |
|  |  |  |  |  |  |
| n-3 | ALA | 0.16 (0.11, 0.21) | 0.16 (0.11, 0.24) | 0.15 (0.11, 0.21) | 0.75 |
|  | EPA | 0.18 (0.13, 0.24) | 0.18 (0.13, 0.24) | 0.18 (0.13, 0.24) | 0.89 |
|  | DPA n-3 | 0.55 (0.47, 0.67) | 0.54 (0.46, 0.67) | 0.56 (0.47, 0.67) | 0.72 |
|  | DHA | 2.53 (2.18, 2.96) | 2.42 (2.09, 2.76) | 2.60 (2.24, 3.03) | **≤0.01** |
|  | Total n-3^e^ | 3.47 (3.08, 3.95) | 3.30 (2.98, 3.78) | 3.51 (3.12, 4.00) | **≤0.05** |
|  | O3I | 2.70 (2.36, 3.17) | 2.58 (2.29, 3.03) | 2.74 (2.41, 3.20) | **≤0.01** |
|  |  |  |  |  |  |
| n-6 | LA | 20.7 (19.4, 21.7) | 20.8 (19.7, 22.1) | 20.6 (19.2, 21.5) | 0.14 |
|  | Linoelaidic | 0.23 (0.20, 0.28) | 0.23 (0.20, 0.27) | 0.23 (0.20, 0.28) | 0.95 |
|  | GLA | 0.15 (0.12, 0.20) | 0.16 (0.12, 0.19) | 0.15 (0.11, 0.20) | 0.74 |
|  | EDA | 0.29 (0.24, 0.33) | 0.28 (0.24, 0.34) | 0.29 (0.25, 0.33) | 0.41 |
|  | DGLA | 1.36 (1.18, 1.52) | 1.35 (1.18, 1.49) | 1.37 (1.19, 1.54) | 0.32 |
|  | AA | 11.0 (9.94, 11.9) | 10.8 (9.60, 11.4) | 11.2 (10.0, 11.9) | **≤0.01** |
|  | DTA | 1.68 (1.44, 1.92) | 1.62 (1.36, 1.80) | 1.72 (1.48, 1.95) | **≤0.01** |
|  | DPA n-6 | 0.58 (0.47, 0.69) | 0.54 (0.46, 0.68) | 0.59 (0.48, 0.70) | 0.06 |
|  | Total n-6^f^ | 36.0 (34.4, 37.4) | 35.8 (34.5, 36.7) | 36.1 (34.3, 37.6) | 0.10 |
|  |  |  |  |  |  |
| Ratios | GLA/LA | 0.01 (0.01, 0.01) | 0.01 (0.01, 0.01) | 0.01 (0.01, 0.01) | 0.93 |
|  | EDA/LA | 0.01 (0.01, 0.02) | 0.01 (0.01, 0.02) | 0.01 (0.01, 0.02) | 0.15 |
|  | DGLA/LA | 0.07 (0.06, 0.08) | 0.07 (0.06, 0.07) | 0.07 (0.06, 0.08) | 0.11 |
|  | AA/DGLA | 8.05 (7.21, 8.99) | 7.88 (7.04, 8.79) | 8.11 (7.31, 9.08) | 0.19 |
|  | T/T | 0.01 (0.01, 0.02) | 0.01 (0.01, 0.02) | 0.01 (0.01, 0.02) | 0.81 |
| ^a^Values represent blood fatty acid (FA) % composition. Stunted defined by height-for-age z-score (HAZ)≤-2. SFA, saturated FA; n-9, omega-9; n-7, omega-7; n-3, omega-3; ALA, alpha-linolenic acid; EPA, eicosapentaenoic acid; DPA n-3, omega-3 docosapentaenoic acid; DHA, docosahexaenoic acid; O3I, omega-3 index; n-6, omega-6; LA, linoleic acid; GLA, gamma-linolenic acid; EDA, eicosadienoic acid; DGLA, dihomo-gamma-linolenic acid; AA, arachidonic acid; DTA, docosatetraenoic acid; DPA n-6, omega-6 docosapentaenoic acid; T/T, triene to tetraene. ^b^P-value from Wilcoxon-Mann-Whitney test comparing stunted and non-stunted children. ^c^Total SFA includes myristic, palmitic, arachidic, behenic, and lignoceric. ^d^Total n-9 includes oleic, elaidic, eicosenoic, Mead, and Nervonic. ^e^Total n-3 includes ALA, EPA, DPA n-3, and DHA. ^f^Total n-6 includes LA, linoelaidic, GLA, EDA, DGLA, AA, DTA, and DPA n-6. | | | | | |
