# Supplementary material for: Association of whole blood n-6 fatty acids with stunting in 2-to-6-year-old Northern Ghanaian children: A cross-sectional study
Source: PLoS One. 2018 Mar 1;13(3):e0193301. doi: 10.1371/journal.pone.0193301 (PMC5832227; doi:10.1371/journal.pone.0193301)
Supplement: S2 Table — Correlations for all FA and growth parameters shown that are significant. (DOCX) [file pone.0193301.s002.docx]

S2 Table: Significant (p≤.05) correlations between all FAs and growth parameters.^a^

|  | Height | Weight | Hb | HAZ | WHZ | BAZ | WAZ | Myristic | Palmitic | Stearic | Arachidic | Behenic | Total SFA^b^ | Oleic | Elaidic | Eicosenoic | Mead | Nervonic | Palmitoleic | Total n-9^c^ | Palmitelaidic | ALA | EPA | DPA n-3 | DHA | Total n-3^d^ | O3I | LA | Linoelaidic | GLA | EDA | DGLA | AA | DTA | DPA n-6 | Total n-6^e^ | GLA/LA | EDA/LA | DGLA/LA | AA/DGLA | T/T |
| --- | --- | --- | --- | --- | --- | --- | --- | --- | --- | --- | --- | --- | --- | --- | --- | --- | --- | --- | --- | --- | --- | --- | --- | --- | --- | --- | --- | --- | --- | --- | --- | --- | --- | --- | --- | --- | --- | --- | --- | --- | --- |
| Age | **0.84** | **0.76** | **0.26** |  |  | **-0.13** |  | **-0.2** | **-0.23** |  | **-0.12** |  | **-0.21** |  | **-0.14** | **-0.26** |  |  | **-0.16** |  |  |  |  |  |  |  |  |  |  | **0.16** |  | **0.12** | **0.16** | **0.11** |  | **0.23** | **0.12** |  |  |  | **-0.12** |
| Height |  | **0.92** | **0.32** | **0.53** |  | **-0.16** | **0.34** | **-0.22** | **-0.22** |  | **-0.13** |  | **-0.17** | **-0.13** |  | **-0.26** |  |  | **-0.19** | **-0.14** |  |  |  |  |  |  |  |  |  | **0.13** |  | **0.17** | **0.24** | **0.21** | **0.14** | **0.26** |  |  | **0.12** |  |  |
| Weight |  |  | **0.29** | **0.5** | **0.34** | **0.21** | **0.55** | **-0.19** | **-0.24** | **0.13** |  |  | **-0.19** |  |  | **-0.24** |  |  | **-0.19** |  |  |  |  |  | **0.12** |  |  |  |  | **0.12** |  | **0.16** | **0.2** | **0.2** |  | **0.22** |  |  | **0.12** |  |  |
| Hb |  |  |  | **0.2** |  |  | **0.12** | **-0.19** | **-0.14** |  |  |  |  | **-0.12** |  | **-0.18** | **-0.12** |  | **-0.16** | **-0.12** |  |  |  |  | **0.26** | **0.25** | **0.24** |  |  |  | **-0.21** |  |  |  |  |  |  | **-0.22** |  |  | **-0.17** |
| HAZ |  |  |  |  |  | **-0.12** | **0.78** |  |  |  |  |  |  | **-0.13** |  |  |  |  | **-0.11** | **-0.12** |  |  |  |  |  |  |  |  |  |  |  |  | **0.18** | **0.2** |  |  |  |  | **0.12** |  |  |
| WHZ |  |  |  |  |  | **0.97** | **0.62** |  |  |  | **0.12** |  |  |  |  |  |  |  |  |  |  |  |  |  |  |  |  |  |  |  |  |  |  |  |  |  |  |  |  |  |  |
| BAZ |  |  |  |  |  |  | **0.49** |  |  |  | **0.13** |  |  |  |  |  |  |  |  |  |  |  |  |  |  |  |  |  |  |  |  |  |  |  |  |  |  |  |  |  |  |
| WAZ |  |  |  |  |  |  |  |  |  |  |  |  |  |  |  |  | **0.12** |  | **-0.12** |  |  |  |  |  |  |  |  |  |  |  |  |  | **0.12** | **0.16** |  |  |  |  |  |  |  |
| Myristic |  |  |  |  |  |  |  |  | **0.32** | **-0.18** | **-0.1** | **-0.34** | **0.22** | **0.23** | **0.16** |  |  | **-0.13** | **0.62** | **0.24** | **0.14** |  |  |  |  |  |  | **-0.21** | **-0.16** | **0.27** | **0.12** |  | **-0.35** | **-0.19** |  | **-0.41** | **0.3** | **0.2** |  | **-0.25** | **0.2** |
| Palmitic |  |  |  |  |  |  |  |  |  | **-0.72** | **-0.33** | **-0.31** | **0.41** | **0.19** |  | **-0.16** |  |  | **0.34** | **0.19** |  | **0.16** |  |  | **-0.19** | **-0.15** | **-0.18** |  | **0.12** |  | **-0.13** | **-0.12** | **-0.41** | **-0.32** | **-0.25** | **-0.4** |  |  |  | **-0.25** | **0.21** |
| Stearic |  |  |  |  |  |  |  |  |  |  | **0.29** | **0.25** | **0.2** | **-0.33** |  | **0.18** |  | **0.16** | **-0.28** | **-0.34** |  | **-0.26** |  | **0.22** | **0.29** | **0.23** | **0.27** | **-0.28** |  |  | **0.21** | **0.18** | **0.52** | **0.5** | **0.36** | **0.23** |  | **0.3** | **0.29** | **0.27** | **-0.22** |
| Arachidic |  |  |  |  |  |  |  |  |  |  |  | **0.56** |  |  |  | **0.44** |  | **0.18** | **-0.27** |  |  |  |  | **-0.15** |  | **-0.14** |  |  |  | **-0.15** | **0.18** | **-0.14** |  |  |  |  | **-0.13** | **0.2** |  | **0.2** |  |
| Behenic |  |  |  |  |  |  |  |  |  |  |  |  | **0.18** | **-0.34** |  | **0.3** |  | **0.66** | **-0.33** | **-0.29** |  | **-0.17** |  |  | **0.14** | **0.12** | **0.16** |  | **0.13** | **-0.18** | **0.11** |  | **0.3** | **0.22** |  | **0.19** | **-0.16** | **0.12** |  | **0.14** |  |
| Total SFA^b^ |  |  |  |  |  |  |  |  |  |  |  |  |  | **-0.21** |  |  |  | **0.39** |  | **-0.19** |  | **-0.12** |  | **0.18** |  |  |  | **-0.54** | **0.13** |  |  |  |  | **0.15** |  | **-0.3** |  | **0.24** | **0.26** |  |  |
| Oleic |  |  |  |  |  |  |  |  |  |  |  |  |  |  |  | **-0.13** | **0.21** | **-0.52** | **0.19** | **0.99** | **-0.14** | **0.13** | **-0.25** | **-0.44** | **-0.49** | **-0.51** | **-0.5** | **-0.19** |  | **0.12** | **-0.31** | **-0.46** | **-0.68** | **-0.45** | **-0.37** | **-0.74** | **0.15** | **-0.22** | **-0.33** |  | **0.5** |
| Elaidic |  |  |  |  |  |  |  |  |  |  |  |  |  |  |  | **0.17** |  |  | **0.19** |  | **0.15** |  | **0.17** | **0.18** |  |  |  |  | **0.13** |  |  |  |  |  |  |  |  |  |  |  |  |
| Eicosenoic |  |  |  |  |  |  |  |  |  |  |  |  |  |  |  |  |  |  |  | **-0.13** |  | **-0.13** | **0.15** |  |  |  |  |  |  | **-0.14** | **0.61** |  |  | **0.14** |  |  |  | **0.61** |  |  | **-0.14** |
| Mead |  |  |  |  |  |  |  |  |  |  |  |  |  |  |  |  |  |  | **0.26** | **0.25** |  |  | **0.12** |  | **-0.16** |  | **-0.13** | **-0.37** | **0.18** | **0.44** |  | **0.37** |  | **0.13** | **0.13** | **-0.23** | **0.5** |  | **0.48** | **-0.37** | **0.88** |
| Nervonic |  |  |  |  |  |  |  |  |  |  |  |  |  |  |  |  |  |  |  | **-0.44** |  |  | **0.22** | **0.28** | **0.27** | **0.29** | **0.29** | **-0.16** |  |  | **0.15** | **0.26** | **0.31** | **0.27** | **0.29** | **0.18** |  | **0.2** | **0.28** |  |  |
| Palmitoleic |  |  |  |  |  |  |  |  |  |  |  |  |  |  |  |  |  |  |  | **0.22** | **0.15** |  | **0.15** | **0.21** |  |  |  | **-0.28** |  | **0.46** |  | **0.17** | **-0.27** | **-0.14** |  | **-0.38** | **0.5** | **0.18** | **0.24** | **-0.4** | **0.36** |
| Total n-9^c^ |  |  |  |  |  |  |  |  |  |  |  |  |  |  |  |  |  |  |  |  | **-0.15** | **0.13** | **-0.23** | **-0.42** | **-0.48** | **-0.5** | **-0.49** | **-0.22** |  | **0.12** | **-0.32** | **-0.45** | **-0.69** | **-0.45** | **-0.36** | **-0.76** | **0.16** | **-0.22** | **-0.31** |  | **0.53** |
| Palmitelaidic |  |  |  |  |  |  |  |  |  |  |  |  |  |  |  |  |  |  |  |  |  |  |  | **0.2** | **0.11** | **0.12** | **0.11** |  |  |  |  |  |  |  | **0.16** |  |  |  |  |  |  |
| ALA |  |  |  |  |  |  |  |  |  |  |  |  |  |  |  |  |  |  |  |  |  |  |  | **-0.2** | **-0.39** | **-0.23** | **-0.37** | **0.32** |  |  |  | **-0.15** | **-0.26** | **-0.2** | **-0.24** |  |  | **-0.14** | **-0.26** |  |  |
| EPA |  |  |  |  |  |  |  |  |  |  |  |  |  |  |  |  |  |  |  |  |  |  |  | **0.5** | **0.35** | **0.52** | **0.46** |  | **0.18** | **0.2** | **0.15** | **0.27** |  | **-0.21** | **-0.19** |  | **0.18** | **0.12** | **0.22** | **-0.23** |  |
| DPA n-3 |  |  |  |  |  |  |  |  |  |  |  |  |  |  |  |  |  |  |  |  |  |  |  |  | **0.54** | **0.71** | **0.58** | **-0.17** |  |  | **0.13** | **0.36** | **0.26** | **0.2** | **0.17** |  |  | **0.2** | **0.39** |  |  |
| DHA |  |  |  |  |  |  |  |  |  |  |  |  |  |  |  |  |  |  |  |  |  |  |  |  |  | **0.94** | **0.99** | **-0.14** |  |  |  | **0.27** | **0.39** |  | **0.17** | **0.16** |  |  | **0.29** |  | **-0.33** |
| Total n-3^d^ |  |  |  |  |  |  |  |  |  |  |  |  |  |  |  |  |  |  |  |  |  |  |  |  |  |  | **0.97** |  |  |  |  | **0.31** | **0.34** |  |  | **0.16** |  |  | **0.31** |  | **-0.27** |
| O3I |  |  |  |  |  |  |  |  |  |  |  |  |  |  |  |  |  |  |  |  |  |  |  |  |  |  |  | **-0.12** |  |  |  | **0.29** | **0.37** |  | **0.13** | **0.16** |  |  | **0.3** |  | **-0.3** |
| LA |  |  |  |  |  |  |  |  |  |  |  |  |  |  |  |  |  |  |  |  |  |  |  |  |  |  |  |  |  |  |  |  | **-0.2** | **-0.26** | **-0.3** | **0.54** | **-0.3** | **-0.31** | **-0.45** |  | **-0.28** |
| Linoelaidic |  |  |  |  |  |  |  |  |  |  |  |  |  |  |  |  |  |  |  |  |  |  |  |  |  |  |  |  |  |  |  |  |  |  |  |  |  |  |  |  | **0.15** |
| GLA |  |  |  |  |  |  |  |  |  |  |  |  |  |  |  |  |  |  |  |  |  |  |  |  |  |  |  |  |  |  |  | **0.34** |  |  | **0.13** |  | **0.98** |  | **0.34** | **-0.42** | **0.44** |
| EDA |  |  |  |  |  |  |  |  |  |  |  |  |  |  |  |  |  |  |  |  |  |  |  |  |  |  |  |  |  |  |  | **0.17** | **0.25** | **0.26** | **0.29** | **0.29** |  | **0.92** | **0.13** |  | **-0.23** |
| DGLA |  |  |  |  |  |  |  |  |  |  |  |  |  |  |  |  |  |  |  |  |  |  |  |  |  |  |  |  |  |  |  |  | **0.4** | **0.33** | **0.33** | **0.36** | **0.34** | **0.2** | **0.91** | **-0.64** | **0.14** |
| AA |  |  |  |  |  |  |  |  |  |  |  |  |  |  |  |  |  |  |  |  |  |  |  |  |  |  |  |  |  |  |  |  |  | **0.67** | **0.59** | **0.63** |  | **0.3** | **0.43** | **0.36** | **-0.39** |
| DTA |  |  |  |  |  |  |  |  |  |  |  |  |  |  |  |  |  |  |  |  |  |  |  |  |  |  |  |  |  |  |  |  |  |  | **0.65** | **0.44** |  | **0.35** | **0.41** | **0.21** | **-0.19** |
| DPA n-6 |  |  |  |  |  |  |  |  |  |  |  |  |  |  |  |  |  |  |  |  |  |  |  |  |  |  |  |  |  |  |  |  |  |  |  | **0.34** | **0.18** | **0.38** | **0.42** | **0.15** | **-0.15** |
| Total n-6^e^ |  |  |  |  |  |  |  |  |  |  |  |  |  |  |  |  |  |  |  |  |  |  |  |  |  |  |  |  |  |  |  |  |  |  |  |  | **-0.19** |  | **0.11** | **0.16** | **-0.51** |
| GLA/LA |  |  |  |  |  |  |  |  |  |  |  |  |  |  |  |  |  |  |  |  |  |  |  |  |  |  |  |  |  |  |  |  |  |  |  |  |  | **0.11** | **0.42** | **-0.4** | **0.48** |
| EDA/LA |  |  |  |  |  |  |  |  |  |  |  |  |  |  |  |  |  |  |  |  |  |  |  |  |  |  |  |  |  |  |  |  |  |  |  |  |  |  | **0.3** |  |  |
| DGLA/LA |  |  |  |  |  |  |  |  |  |  |  |  |  |  |  |  |  |  |  |  |  |  |  |  |  |  |  |  |  |  |  |  |  |  |  |  |  |  |  | **-0.53** | **0.24** |
| AA/DGLA |  |  |  |  |  |  |  |  |  |  |  |  |  |  |  |  |  |  |  |  |  |  |  |  |  |  |  |  |  |  |  |  |  |  |  |  |  |  |  |  | **-0.5** |

^a^Hb, hemoglobin; HAZ, height-for-age z-score; WHZ, weight-for-height z-score; BAZ, BMI-for-age z-score; WAZ, weight-for-age z-score; SFA, saturated fatty acid; n-9, omega-9; ALA, alpha-linolenic acid; EPA, eicosapentaenoic acid; DPA n-3, omega-3 docosapentaenoic acid; DHA, docosahexaenoic acid; n-3, omega-3; O3I, omega-3 index; LA, linoleic acid; GLA, gamma-linolenic acid; EDA, eicosadienoic acid; DGLA, dihomo-gamma-linolenic acid; AA, arachidonic acid; DTA, docosatetraenoic acid; n-6, omega-6; T/T, triene to tetraene ratio. ^b^Total SFA includes myristic, palmitic, arachidic, behenic, and lignoceric. ^c^Total n-9 includes oleic, elaidic, eicosenoic, Mead, and Nervonic. ^d^Total n-3 includes ALA, EPA, DPA n-3, and DHA. ^e^Total n-6 includes LA, linoelaidic, GLA, EDA, DGLA, AA, DTA, and DPA n-6.
