# Supplementary material for: Association of whole blood n-6 fatty acids with stunting in 2-to-6-year-old Northern Ghanaian children: A cross-sectional study
Source: PLoS One. 2018 Mar 1;13(3):e0193301. doi: 10.1371/journal.pone.0193301 (PMC5832227; doi:10.1371/journal.pone.0193301)
Supplement: S3 Table — Model is not adjusted for sex as there were few significantly different FAs between sexes and regression values were essentially unaffected when evaluated with sex adjustment. (DOCX) [file pone.0193301.s003.docx]

| S3 Table: Regression results between HAZ, WAZ, and fatty acids (model: HAZ= fatty acid + hemoglobin; WAZ= fatty acid + hemoglobin; HAZ= ratio + hemoglobin; WAZ= ratio + hemoglobin).^a^ | | | | | | | | | | |
| --- | --- | --- | --- | --- | --- | --- | --- | --- | --- | --- |
|  |  | **HAZ** | | | | **WAZ** | | | |  |
| **Class** | **Fatty acid** | **Beta ± SE** | **p-value** | | | **Beta ± SE** | **p-value** | | |  |
| SFA | Myristic | -0.47 ± 0.56 | 0.41 | | | -0.16 ± 0.43 | 0.71 | | |  |
|  | Palmitic | -0.01 ± 0.04 | | 0.72 | -0.05 ± 0.03 | | | 0.08 |  |  |
|  | Stearic | 0.05 ± 0.04 | | 0.25 | 0.07 ± 0.03 | | | **≤0.05** |  |  |
|  | Arachidic | -0.86 ± 0.96 | | 0.37 | 0.48 ± 0.74 | | | 0.52 | |  |
|  | Behenic | 0.01 ± 0.35 | 0.98 | | | 0.22 ± 0.27 | | 0.40 | |  |
|  | Lignoceric | -0.06 ± 0.20 | 0.76 | | | 0.02 ± 0.15 | | 0.89 | |  |
|  | Total SFA^b^ | 0.03 ± 0.05 | 0.54 | | | 0.01 ± 0.04 | 0.84 | | |  |
|  |  |  |  | | |  |  | | |  |
| n-9 | Oleic | -0.04 ± 0.02 | 0.10 | | | -0.03 ± 0.02 | 0.14 | | |  |
|  | Elaidic | -0.02 ± 0.33 | 0.94 | | | -0.04 ± 0.25 | 0.87 | | |  |
|  | Eicosenoic | -0.35 ± 0.60 | 0.56 | | | -0.01 ± 0.46 | 0.98 | | |  |
|  | Mead | 2.48 ± 1.21 | **≤0.05** | | | 2.08 ± 0.93 | **≤0.05** | | |  |
|  | Nervonic | 0.13 ± 0.29 | 0.65 | | | 0.14 ± 0.23 | 0.53 | | |  |
|  | Palmitoleic | -0.37 ± 0.32 | 0.25 | | | -0.40 ± 0.24 | 0.10 | | |  |
|  | Total n-9^c^ | -0.04 ± 0.02 | 0.10 | | | -0.03 ± 0.02 | 0.15 | | |  |
|  |  |  |  | | |  |  | | |  |
| n-7 | Palmitelaidic | 3.13 ± 2.47 | 0.21 | | | 2.15 ± 1.90 | 0.26 | | |  |
|  |  |  |  | | |  |  | | |  |
| n-3 | ALA | -0.67 ± 0.56 | 0.23 | | | -0.32 ± 0.43 | 0.45 | | |  |
|  | EPA | 0.07 ± 0.25 | 0.77 | | | 0.17 ± 0.19 | 0.36 | | |  |
|  | DPA n-3 | 0.03 ± 0.37 | 0.93 | | | 0.20 ± 0.28 | 0.48 | | |  |
|  | DHA | 0.08 ± 0.10 | 0.43 | | | 0.09 ± 0.08 | 0.26 | | |  |
|  | Total n-3^d^ | 0.04 ± 0.07 | 0.62 | | | 0.06 ± 0.06 | 0.27 | | |  |
|  | O3I | 0.06 ± 0.08 | 0.47 | | | 0.08 ± 0.06 | 0.23 | | |  |
|  |  |  |  | | |  |  | | |  |
| n-6 | LA | -0.06 ± 0.03 | 0.07 | | | -0.04 ± 0.03 | 0.11 | | |  |
|  | Linoelaidic | 1.44 ± 0.99 | 0.15 | | | 0.16 ± 0.76 | 0.83 | | |  |
|  | GLA | -0.19 ± 0.95 | 0.84 | | | -0.21± 0.73 | 0.78 | | |  |
|  | EDA | -1.48 ± 0.94 | 0.12 | | | -0.80 ± 0.72 | 0.27 | | |  |
|  | DGLA | 0.51 ± 0.25 | **≤0.05** | | | 0.39 ± 0.19 | **≤0.05** | | |  |
|  | AA | 0.12 ± 0.04 | **≤0.01** | | | 0.08 ± 0.03 | **≤0.01** | | |  |
|  | DTA | 0.50 ± 0.17 | **≤0.01** | | | 0.39 ± 0.13 | **≤0.01** | | |  |
|  | DPA n-6 | 0.56 ± 0.38 | 0.14 | | | 0.18 ± 0.29 | 0.54 | | |  |
|  | Total n-6^e^ | 0.03 ± 0.03 | 0.18 | | | 0.02 ± 0.02 | 0.26 | | |  |
|  |  |  |  | | |  |  | | |  |
| Ratios | GLA/LA | 4.19 ± 17.7 | 0.81 | | | 0.54 ± 13.6 | 0.97 | | |  |
|  | EDA/LA | -13.8 ± 18.3 | 0.45 | | | -6.52 ± 14.0 | 0.64 | | |  |
|  | DGLA/LA | 11.7 ± 4.39 | **≤0.01** | | | 8.34 ± 3.38 | **≤0.01** | | |  |
|  | AA/DGLA | 0.02 ± 0.04 | 0.68 | | | 0.00 ± 0.03 | 0.92 | | |  |
|  | T/T | 11.3 ± 12.1 | 0.35 | | | 9.75 ± 9.28 | 0.29 | | |  |
| ^a^Model is not adjusted for sex as there were few significantly different FAs between sexes and regression values were essentially unaffected when evaluated with sex adjustment. HAZ, height-for-age z-score; WAZ, weight-for-age z-score; SFA, saturated fatty acid; n-9, omega-9; n-7, omega-7; n-3, omega-3; ALA, alpha-linolenic acid; EPA, eicosapentaenoic acid; DPA n-3, omega-3 docosapentaenoic acid; DHA, docosahexaenoic acid; O3I, omega-3 index; n-6, omega-6; LA, linoleic acid; GLA, gamma-linolenic acid; EDA, eicosadienoic acid; DGLA, dihomo-gamma-linolenic acid; AA, arachidonic acid; DTA, docosatetraenoic acid; DPA n-6, omega-6 docosapentaenoic acid; T/T, triene to tetraene ratio.  ^b^Total SFA includes myristic, palmitic, arachidic, behenic, and lignoceric. ^c^Total n-9 includes oleic, elaidic, eicosenoic, Mead, and Nervonic. ^d^Total n-3 includes ALA, EPA, DPA n-3, and DHA. ^e^Total n-6 includes LA, linoelaidic, GLA, EDA, DGLA, AA, DTA, and DPA n-6. | | | | | | | | | | |
